# Les chambres à cathéters implantables: à propos d’une série de 970 cas

**Published:** 2012-07-13

**Authors:** Hassane El Kabiri, Massine El Hammoumi, Akram Traibi, Faycal El Oueriachi, Adil Arsalane

**Affiliations:** 1 Service de Chirurgie Thoracique, Hôpital Militaire d’Instruction Mohammed V, Faculté de Médecine et de Pharmacie, Université Mohamed V- Souisi, Rabat, Maroc

**Keywords:** Cathéter implantable, technique de pose, complications, implantable catheters, implementation technic, complications

## Abstract

Notre étude a pour but d’évaluer les indications, les techniques, ainsi que les complications de la pose des chambres à cathéters implantables et leur utilisation à long terme. Il s’agit d’une étude rétrospective sur une période de 8 ans (Septembre 2002-Septembre 2010); 970 patients ont bénéficié de la pose d’une chambre à cathéter implantable, avec 405 hommes et 565 femmes, d’âge moyen de 46,8 ans. La pose a été réalisée par voie chirurgicale, ou par voie percutanée. Les indications étaient celles d’une chimiothérapie ou d’une antibiothérapie (10 cas), tous les patients ont eu un contrôle radioscopique dans la salle d’intervention et d’un document radiographique post-opératoire. La voie d’abord a été chirurgicale dans 352 cas et par voie percutanée dans 618 des cas dont 533 par ponctions de la veine sous-clavière. Le côté droit a été utilisé de préférence dans 843 cas et 127 poses à gauche. Les incidents per opératoires ont été sous formes des difficultés de pose (34 cas) d’hématome sous-cutanée (17 cas) et un cas pneumothorax. Les complications à court ou à long terme ont été présente dans 5,25% des cas avec 15 cas d’infection du site opératoire, 3 sepsis, 19 cas de thromboses veineuses, 5 cas d’obturation, 5 cas de nécrose cutanée et 4 migrations du cathéter dans la veine jugulaire (n=1) et la veine cave supérieure (n=1) et les cavités cardiaques (2 cas). La pose d’une chambre implantable est souvent réalisé pour une chimiothérapie anticancéreuse rarement pour une antibiothérapie au long court; le choix d’une technique, le maniement des chambres doit répondre à une certaines rigueur dans le respect des règles de l’asepsie et une bonne prise en charge diagnostic des anomalies survenant lors de l’utilisation des dispositifs.

## Introduction

Les chambres à cathéters implantables (CCI) sont apparues il y a une quinzaine d’années. Elles se sont rapidement imposées comme un outil essentiel dans la prise en charge des patients nécessitant l’administration de chimiothérapies intensives et/ou prolongées, la nutrition parentérale, l’antibiothérapie et la transfusion de dérivés sanguins. L’administration des cytotoxiques constitue l’indication la plus fréquente. Nous rapportons une expérience concernant la pose et le maniement des sites implantables à propos d’une série de 970 cas.

## Méthodes

Il s’agit d’une étude rétrospective sur une durée de 8 ans de Septembre 2002- Septembre 2010. La réalisation du geste a été programmée, les patients ont été transférés dans notre formation de divers services et pour chaque pose nous avons établi une fiche sur laquelle tous les renseignements ont été notés. Tous nos patients ont été informés en leur expliquant l’indication et le matériel à utiliser.

L’indication principale de la pose a été principalement une chimiothérapie anti-cancéreuse dont les étiologies sont représentées dans le Tableau 1. La pose des chambres à cathéters implantables a été réalisée au bloc opératoire dans les conditions opératoires d’asepsie et sous anesthésie locale à la Lidocaine 5%.

**Tableau 1:** Les étiologies imposant la mise des chambres à cathéter implantables

	Nombre	%
Cancers gynécologiques	56	5,7%
Cancer du sein	245	25,3%
Cancers digestifs	150	15,5%
Cancers broncho-pulmonaires	185	19,1%
Lymphomes et leucémies	244	25,2%
Cancers ORL	67	6,9%
Cancers ostéo-articulaires	13	1,4%
Antibiothérapies au long court pour ostéites septiques	10	1,03%

**Figure 1:**
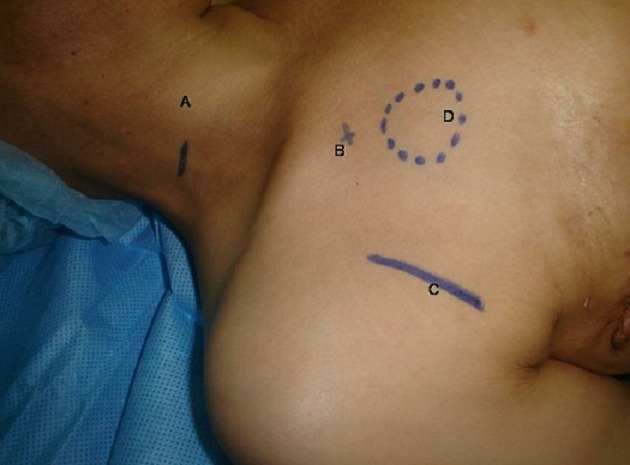
Sites de ponction A: veine jugulaire interne, B: veine sous clavière, C: veine céphalique, D: Emplacement de la chambre

La technique par préférence n’a répondu a aucun critère et deux voies ont été utilisée soit un abord par dissection chirurgicale de la veine céphalique ou de la veine jugulaire externe, ou bien la voie transcutanée par ponction de la veine sous-clavière ou de la veine jugulaire interne (Figure 1), la présence d’un reflux sanguin veineux et d’un bon passage après injection ont été les critères de la réussite de la pose. Une radioscopie au bloc a été faite pour tous les patients avec retrait d’un cliché radiographique montrant l’emplacement du cathéter et de la chambre. Tous les patients ont séjournée pendant au moins 24 heures avant leur transfert à leur service d’origine. Nous n’avons pas utilisé d’antibioprophylaxie systématique pour nos patients.

## Résultats

L’abord veineux a été réalisé dans 352 cas par dissection chirurgicale (dans 302 cas la veine céphalique a été abordée par le sillon delto-pectoral et dans 50 cas c’était la veine jugulaire externe) et dans 618 cas la pose a été réalisée par voie transcutanée avec une veine sous-clavière dans 533 cas et une veine jugulaire interne dans 85 cas. Le côté droit a été utilisé de préférence chez 843 patients contre 127 poses à gauche (Tableau 2).

**Tableau 2:** Technique et coté de pose

		Droit	Gauche
Voie chirurgicale (352 cas)	Céphalique (294 cas)	272	22
	Jugulaire externe (58 cas)	42	16
Voie transcutanée (618 cas)	Sous-clavière (561 cas)	481	80
	Jugulaire interne (57 cas)	48	09

L’échec de pose du cathéter par voie céphalique a été en rapport avec une veine trop grêle (25 cas) ou une impossibilité de progression du cathéter (6 cas). Dans ces cas un abord de la veine jugulaire externe ou un changement de la technique avec abord percutanée a été réalisé. L’artère sous-clavière a été ponctionnée 9 fois/561 dans notre série correspondant à un pourcentage de 1,6% et un cas de pneumothorax a été observé (0,2%). En post-opératoire immédiat nos patients ont été hospitalisés pendant au moins 24 heures avec survenue d’un hématome sous-cutanée dans 24 cas traités par pansement compressif dans 12 cas et ayant nécessité une reprise chirurgicale pour hémostase dans 5 cas (0,5%).

Les Complications liées à l’utilisation de la chambre à court et à et à long terme ont été retrouvés dans 5,25% (51 cas) dont 1,5% (15 cas) après la voie chirurgicale et 3,7% (36 cas) après la voie percutanée.

**Figure 2:**
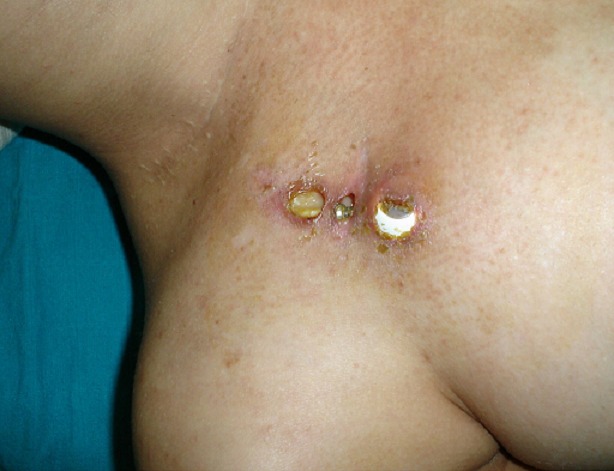
Infection locale cathéter et chambre a nus

L’infection du site opératoire a été survenue entre 2 et 14 mois (moyenne à 6mois) après la pose. Le diagnostic était facile devant la présence de douleurs du site opératoire et la constatation à l’examen clinique de signes locaux d’inflammation (10 cas), ou d’issue de pus (4 cas) et une mise a nu du cathéter (1 cas) (Figure 2). Cette complication a conduit en l’ablation du dispositif et mise sous aux antibiotiques probabiliste en attendant les résultats de l’examen bactériologique dont le seul germe rencontré est le *Staphylocoque aureus* sensible au traitement anti-staphylococcique habituel.

Le sepsis a été présent chez trois patients, dont un cas avec une infection locale associée. Le diagnostic a été posé devant la présence d’un état fébrile persistante une l’ablation du dispositif avec examen bactériologique et instauration d’un traitement antibiotique probabiliste orientée en fonction de l’écologie bactérienne la plus fréquente dans notre contexte et les germes rencontrés étaient *Staphylocoque aureus*, *Staphylocoque epidermidis* et *Pseudomonas aeroginosa* multirésistant. L’évolution était favorable pour deux patients avec apyrexie obtenu après deux jours, et pour le troisième patient dont l’infection a été due au *Pseudomonas aeroginosa*, un choc septique est survenu après 3 jours du diagnostic de sepsis, nécessitant une hospitalisation dans le service de réanimation dont l’évolution était favorable après une antibiothérapie et prise en charge pendant 15 jours.

Les thromboses veineuses révélées par la présence de douleur avec un œdème du bras ont été confirmée par la réalisation d’une écho-doppler dont le siége était la veine sous-clavière à différents niveaux, et la prise en charge a consisté en une héparinothérapie dans 19 cas qui a permis de traiter 5 patients et dans deux cas l’ablation de la chambre a été nécessaire devant une chambre non fonctionnelle. Aucun cas d’embolie pulmonaire n’a été observé chez nos patients.

L’obturation a été observée dans 5 cas jugulé après rinçage à l’héparine diluée au sérum physiologique, et chez 5 patients l’extravasation du produit de contraste avec nécrose cutanée été notée conduisant en l’ablation du dispositif.

**Figure 3:**
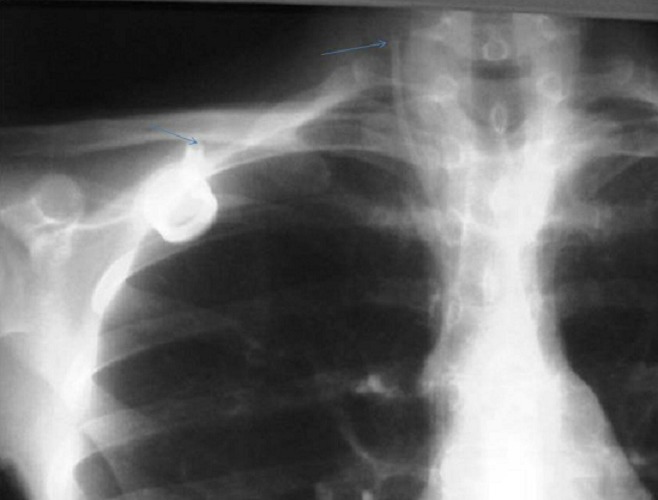
Radiographie thoracique de face on note la migration du cathéter au niveau de la veine jugulaire interne droite loin de la chambre (Flèches: bout du cathéter dans la VJI déconnecté de la chambre)

Les migrations du cathéter par déconnexion ont été diagnostiquées lors d’une radiographie du thorax de face (Figure 3), cette migration a été noté dans la veine jugulaire interne (1 cas) et dont l’ablation était réalisée par abord chirurgical direct. Les autres sites de migrations ont été la veine cave supérieure (1 cas), les cavités cardiaques (2 cas) et l’extraction a été réalisée par voie endo-vasculaire

## Discussion

La chimiothérapie anti-cancéreuse nécessite un abord veineux efficace et pratique et pour cela les chambres à cathéters implantables ont été développées depuis 1980 dont l’utilisation permet l’administration d’agents cytotoxiques en accédant au système veineux central d’une façon permanente et prolongé avec préservation du capital veineux périphérique et le confort des patients est mis en jeu.

La pose peut être réalisée sous anesthésie locale ou générale selon le choix du chirurgien et du patient de chacune [1]. La technique de la pose a été débattu par plusieurs auteurs on analysant les inconvénients et les avantages de chacune d’elle, mais la voie chirurgicale est pour la plupart une référence parce qu’elle est facile, efficace, et sécurisé, elle ne présente pas de contre indication absolue en dehors de l’infection. Mais pour la majorité un temps de phlébo-cavographie est systématique, ce qui n’est pas toujours aisé [1].

La voie percutanée est une alternative intéressante permettant l’accès direct au système veineux profond quoiqu’elle expose au risque de ponction artérielle et pleurale, leur faible incidence décrite dans la littérature ne juge pas de préférer la voie chirurgicale uniquement. Dans notre contexte nous avons initialement commencé par pratiquer la voie chirurgicale et depuis la disponibilité de kit permettant la réalisation de la voie percutanée nous avons utilisé cette voie d’abord sans abandonnée la voie chirurgicale et nous avons remarqué sur les résultats sus décrites qu’il n’y a pas de différence significative entre les deux techniques pendant la période post-opératoire immédiate ou à long terme [2, 3].

La préférence du côté droit (843 cas) pour nous se fait pour des raisons anatomiques et le choix se fait également en fonction des conditions locales, dont certaines contre-indications nous avons préférer la pose du côté gauche (irradiation préalable, infection ou d’anomalie anatomique), la constatation décrites dans la plupart des articles.

L’hématome post-opératoire ne constitue aucun danger vital pour le patient mais représente un inconfort qui nécessite un traitement médical ou chirurgical en cas d’échec. Dans 1,75% (17 cas) un hématome a été diagnostiqué dont 5 cas (0,5%) seulement ont été repris pour hémostase. Cette complication est retrouvée dans 0,9% à 3,6% des cas dans la littérature (Tableau 3) [4–8]. L’infection représente la principale complication survenue lors de l’utilisation des chambres à cathéters implantables, les germes habituellement retrouvés dans la littérature sont les *Staphylococcus aureus* et les Staphylocoques coagulase négative. Nous avons retrouvée un *Staphylococcus aureus* dans 12 cas, un *Staphylococcus epidermidis* dans 2 cas et un cas où le germe isolé était *Pseudomonas aeroginosa*. L’infection survient à distance du bloc opératoire ce qui explique qu’elle est plutôt liée à l’utilisation des chambres qui doit se faire avec des précautions d’asepsie rigoureuse seules garantes d’un maintien plus prolongé et permet de diminuer le taux d’infection. Toutes les infections locales ont conduit à l’ablation du dispositif avec mise en route d’un traitement antibiotique probabiliste puis adapté en fonction des résultats bactériologiques [8, 9].

**Tableau:** Incidence des complications précoces et tardives dans la littérature et dans notre série

	Champault 1985 325 cas	Paoli 1994 164 cas	Boussen 2001 205 cas	Talfer 2003 116 cas	Rouzrokh 2009 524 cas	Goltz 2010 763 cas	Notre série 2010 970 cas
Pneumothorax	–	3,3%	1,4%	–	–	–	0,1%
Hématome	2%	2,6%	2,9%	0,9%	1,1%	–	1,7%
Infection du site opératoire	2,7%	1,2%	–	0,9%	8,8%	5,4%	1,5%
Sepsis	2,7%	0,6%	3,9%	8,6%	–	–	0,3%
Thrombose veineuse	3,6%	1,3%	2,9%	5,2%	6,1%	8,5%	1,9%
Migration du cathéter	0	0,6%	1%	3,4%	6,7%	0,1	0,4%
Obturation	3,6%	1,3%	–	3,4%	6,1%	0,1%	0,5%

Les infections malgré les précautions surviennent au cours de l’utilisation et restent présentes dans la littérature (Tableau 3). Certains auteurs ont introduit la notion de verrou local d’antibiotique dans un but préventif, mais pour la plupart il n’a pas d’intérêt [7, 9, 10].

Les thromboses veineuses profondes représentent la deuxième complication à long terme retrouvée dans la littérature avec une fréquence située entre 1,3% et 6%, mais ces incidences ne reflète pas la réalité de la présence de thrombose veineuse et c’est seulement les thromboses symptomatiques qui sont diagnostiqué, puisque Balesteri et al [11] retrouve 45% de thromboses incomplètes et 10% de thromboses complètes après une évaluation phlébographique systématique, et aussi 60% des thromboses sur cathéter sont asymptomatiques et ne sont diagnostiquées que par l’échographie doppler systématique aboutissant à une incidence globale d’environ 14%. Leur traitement repose sur l’héparinothérapie à dose curative, relayée par antivitamine K. nos patients ont bien évolué après traitement et chez deux patients l’explantation était nécessaire.

La migration du cathéter par déconnexion de la chambre et les cas d’obturation de la chambre à cathéter implantable a été décrite dans la littérature (Tableau 3). L’obturation de la chambre a été retrouvée dans 5,4%, elle est essentiellement dus à des dépôts de fibrine avec formation d’un caillot de sang, nous pratiquant un rinçage à l’héparine diluée dans du sérum physiologique et pour tout les cas le reflux sanguin était positif après cette manœuvre, les auteurs utilise le sérum physiologique uniquement. L’héparinisation dans un but préventif n’a aucun intérêt et elle est abandonnée pour la majorité des auteurs, elle n’a pas fait ses preuves et ne fait l’objet d’aucun consensus. La migration dans la veine cave supérieure ou dans les cavités cardiaques nécessite toujours une extraction par voie endovasculaire. Les différentes incidents et complications rapportées dans la littérature et dans notre série ont été résumés dans le Tableau 3.

## Conclusion

L’implantation d’une chambre à cathéters améliore la prise en charge des patients cancéreux en leur confinant une voie d’administration permanente et prolongée le choix d’une technique ou de l’autre dépend de plusieurs paramètres. Le taux de complications lors de l’utilisation doit être diminué par la bonne information du personnel soignant et médical. La migration est une complication qui doit être évoqué devant toute anomalie de fonctionnement du dispositif et réaliser une radiographie thoracique.
